# Characteristics of Gallium Nitride-Based Dual-Gate Metal-Oxide-Semiconductor High-Electron-Mobility Transistors with Gate Oxide Layers Directly Grown by Photoelectrochemical Oxidation Method

**DOI:** 10.3390/mi17060645

**Published:** 2026-05-24

**Authors:** Zih-Siang Hung, Hsin-Ying Lee, Ricky W. Chuang, Ching-Ting Lee

**Affiliations:** 1 Institute of Microelectronics, Department of Electrical Engineering, National Cheng Kung University, Tainan 701, Taiwan; hermanxiang0203@gmail.com (Z.-S.H.); rwchuang@mail.ncku.edu.tw (R.W.C.); 2 Department of Photonics, National Cheng Kung University, Tainan 701, Taiwan; 3 Department of Electrical Engineering, Yuan Ze University, Taoyuan 320, Taiwan

**Keywords:** dual-gate structure, GaN-based metal-oxide-semiconductor high-electron-mobility transistors, low-frequency noise characteristics, photoelectrochemical oxidation method

## Abstract

To minimize the influence of interface states and surface damage, by inserting a gate oxide layer, the photoelectrochemical oxidation method was utilized to directly grow the gate oxide layer while simultaneously creating the gate-recessed regions onto gallium nitride (GaN)-based single-gate and dual-gate metal-oxide-semiconductor high-electron-mobility transistors (MOS-HEMTs). Compared to the single-gate structure, the two-dimensional electron gas (2DEG) channel layer was also modulated by the auxiliary gate, in addition to being modulated by the main gate. Consequently, a wider transconductance range, larger saturation drain-source current, lower gate leakage current, and higher drain-source breakdown voltage were the benefits derived from the auxiliary gate functionality in the dual-gate devices. Moreover, the low-frequency noise characteristics of the GaN-based MOS-HEMTs could also be improved by the dual-gate structure. These experimental results demonstrated that incorporating a dual-gate structure and directly grown gate oxide layers onto GaN-based MOS-HEMTs is a promising alternative for GaN-based low-noise, high-power, and high-frequency applications.

## 1. Introduction

With the urgently growing demand for energy consumption in high-frequency and high-power systems, highly efficient power-handling electronic devices have recently become an unavoidable requirement. Although silicon- and GaAs-based electronic devices and integrated circuits have become dominant products applied in various systems, gallium nitride (GaN)-based electronic devices and integrated circuits have emerged as a prime substitute for post-Si- and GaAs-based next-generation candidates owing to their inherent wide energy bandgap, high breakdown voltage, and high saturation velocity [1]. Given the advantages of the two-dimensional electron gas (2DEG) induced at the AlGaN/GaN heterostructured interface, Schottky-gate metal-semiconductor high-electron-mobility transistors (MES-HEMTs) were, in turn, developed and utilized [2,3]. However, they were prone to high gate leakage current, trapping effect, current collapse, and low breakdown voltage [4,5,6]. Consequently, the problems associated with the limited gate voltage swing, maximum output power, power handling capability, and long-term reliability adversely curtailed the extent of their applications in various systems. To mitigate the undesired flaws and disadvantages, GaN-based metal-oxide-semiconductor high-electron-mobility transistors (MOS-HEMTs) have been fabricated by inserting various dielectric oxide layers between the GaN-based semiconductor and the gate metal [7,8,9]. However, the performance of the GaN-based MOS-HEMTs tends to be degraded by the induced interface state and the contamination of the inserted gate dielectric oxide layer. To prevent undesired deterioration, the gate oxide layer was directly grown using the photoelectrochemical (PEC) oxidation method, which has been previously employed to oxidize GaN-based semiconductors [10,11]. In general, to improve breakdown voltage by reducing localized electric field spikes, the formation of gate-recessed regions beneath the gate electrode was a widely used method [12]. As an added benefit, the direct gate oxidation route could also simultaneously achieve the gate-recessed regions of the GaN-based MOS-HEMTs using the PEC oxidation method [13,14]. Despite the extensive application of advanced epitaxial growth techniques and innovative manufacturing processes to minimize defects, interface states, and structural damage, thereby improving device performance, the characteristics of the resulting GaN-based MOS-HEMTs remained constrained by their design configuration. In conventional GaN-based MOS-HEMTs, a single-gate (SG) electrode is typically fabricated within the space between the source and drain electrodes. In addition to achieving high power gain and robust breakdown performance, various dual-gate (DG) structures have been proposed for GaN-based MOS-HEMTs to enhance input-to-output isolation [15,16,17,18]. To minimize the impact of interface states and surface damage on devices’ performance, the dual-gate structure enables more precise control and verification, thereby contributing to device optimization. In this work, surface treatment with an (NH_4_)_2_S_x_ (S = 6%) chemical solution was employed to remove the native oxide layer residing on the surface of the epitaxial samples. Additionally, the PEC oxidation method was applied to minimize interface states and surface damage for both gate oxide formation and gate recess fabrication. Single-gate and dual-gate electrode structures, comprising a main gate and an auxiliary gate, were implemented in GaN-based depletion-mode MOS-HEMTs. The characteristics of the fabricated devices were subsequently measured, compared, and analyzed.

## 2. Device Structure and Fabrication

**Figure 1:**
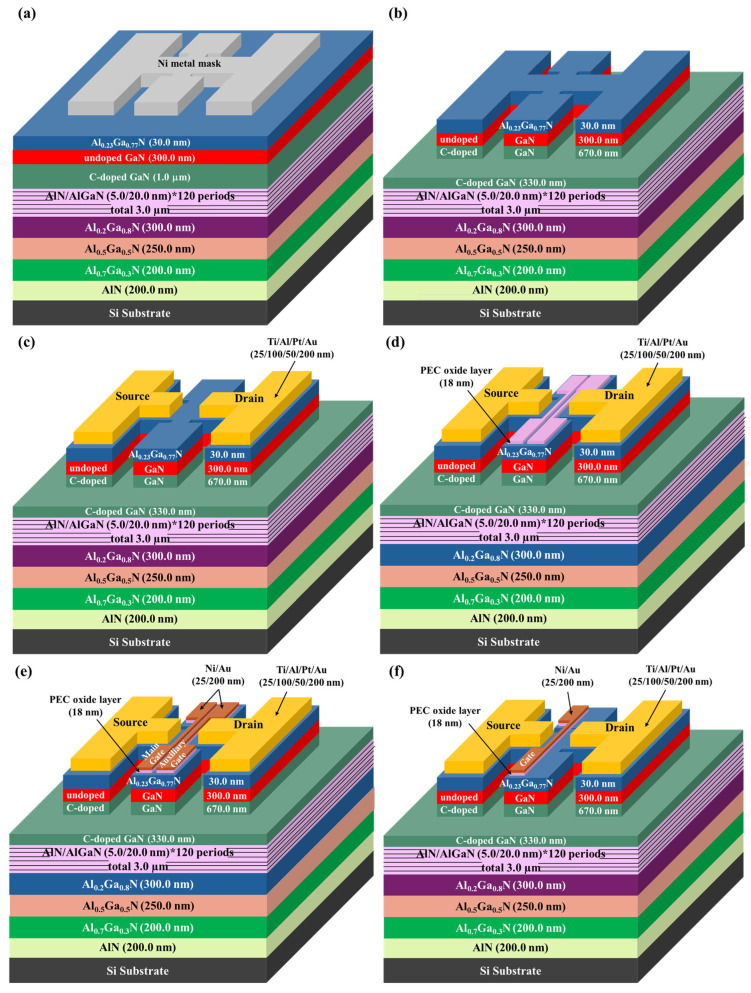
Three-dimensional configurations of GaN-based MOS-HEMTs with (**a**) Ni-metal mask pattern, (**b**) mesa isolation region, (**c**) source electrode and drain electrode, (**d**) gate oxide layer and source and drain electrodes, (**e**) dual-gate structure, and (**f**) single-gate structure.

**Figure 2:**
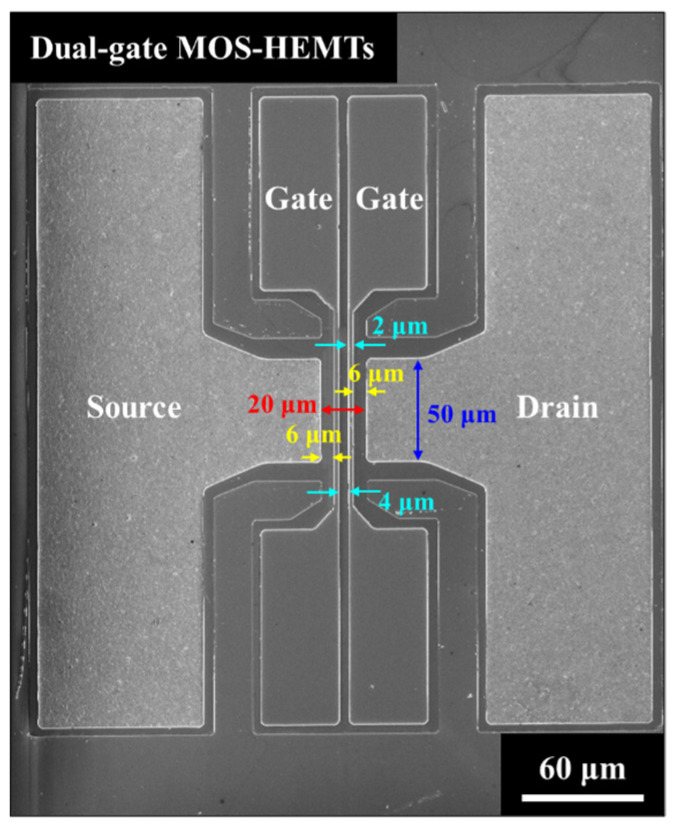
Top view of scanning electron microscopy image of GaN-based dual-gate MOS-HEMTs.

The epitaxial layers used in this study were grown on (111)-oriented p-type silicon (Si) substrates using a Veeco Propel metal-organic chemical vapor deposition (MOCVD) system. At first, the 6-inch Si wafers were chemically cleaned to remove contaminants and native oxide residing on the surface. Because of the presence of a 17% lattice mismatch between Si and GaN, a high-temperature 200.0-nm-thick AlN nucleating layer was grown on the cleaned Si substrates to facilitate lattice compatibility between the Si substrate and the GaN-based layers. Following this growth process, a step-three graded AlGaN buffer layers (Al_0.7_Ga_0.3_N layer (200.0 nm), Al_0.5_Ga_0.5_N layer (250.0 nm), and Al_0.2_Ga_0.8_N layer (300.0 nm)), in which the buffer layers gradually shifted the composition from AlN to GaN, and 120 periodic pairs of AlN/Al_0.2_Ga_0.8_N (5.0 nm/20.0 nm) superlattice layers were grown to manage stress and dislocations arising from lattice mismatch. To form the 2DEG channel, a carbon-doped i-GaN buffer layer (1.0 μm), an undoped i-GaN channel layer (300.0 nm), and an undoped Al_0.23_Ga_0.77_N barrier layer (30.0 nm) (hereafter referred to as an AlGaN layer) were subsequently grown. Room-temperature measurements yield a sheet electron density of 2.0 × 10^13^ cm^−2^ and an electron mobility of 1500 cm^2^/V-s. Device fabrication began with mesa isolation using a standard photolithography method to open the windows of mesa regions, a 100-nm-thick Ni metal was deposited by an electron beam evaporator. The patterned Ni metal mask, as shown in Figure 1a, was obtained by a lifted-off process. The sample was then etched using a BCl_3_ etchant in a reactive ion etching system, etching down to the carbon-doped i-GaN buffer layer. When the residual Ni metal mask was removed using diluted hydrochloric acid, the mesa isolation configuration was determined, as shown in Figure 1b. To completely remove the native oxide residing on the surface of the undoped AlGaN barrier layer, the sample was dipped into an (NH_4_)_2_S_x_ (S = 6%) solution at 60 °C for 30 min [19]. After the windows of the source and drain regions were opened using a standard photolithography system, Ti/Al/Pt/Au (25/100/50/200 nm) stacked metals were then deposited as the source and drain electrodes using an electron-beam evaporator. After the undesired metals were lifted off, the ohmic properties were obtained by thermally annealing them in a nitrogen ambient rapid thermal annealing system at 900 °C for 30 s [20]. The configuration with source and drain electrodes is shown in Figure 1c. The specific contact resistance was approximately 7.0 × 10^−6^ Ω-cm^2^. The separation between the source electrode and drain electrode was 20 μm. The window patterns of SG and DG regions were opened using a standard photolithography method. To completely remove the native oxide and contamination, the samples were cleaned with a (NH_4_)_2_S_x_ (S = 6%) solution again. After that, the samples were dipped into a H_3_PO_4_ electrolyte solution at a pH value of 3.5 and illuminated with a helium–cadmium (He-Cd) laser (power density = 10 mW/cm^2^ and wavelength = 325 nm). Since the photon energy of the He-Cd laser was larger than the bandgap energy of the AlGaN barrier layer, the electron–hole pairs were generated on the surface of the AlGaN barrier layer. The generated holes (h^+^) would be driven to the interface between the H_3_PO_4_ solution and the AlGaN barrier layer by the built-in electric field and the applied direct current (DC) voltage [14,21]. Consequently, the AlGaN barrier layer was oxidized, forming a composite material of Ga_2_O_3_ and Al_2_O_3_. The composition ratio of Ga_2_O_3_ and Al_2_O_3_ was determined by the Al and Ga contents of the AlGaN barrier layer. The PEC oxidation process was expressed as [14]:

(1) $$2\mathrm{AlGaN}\;+\;12h^{+}\;+\;6H_{2}O\;⇄\;{\mathrm{Al}}_{2}O_{3}\;+\;{\mathrm{Ga}}_{2}O_{3}\;+\;N_{2}\;+\;12H^{+}$$

while the oxide layer of composited materials of Ga_2_O_3_ and Al_2_O_3_ was grown, it was also etched by the H_3_PO_4_ electrolytic chemical solution simultaneously [11]. During the PEC oxidation process, the growth and etching of the oxides occurred simultaneously. The net growth rate was approximately 3.7 nm/min. During the growth of the oxide layer, the AlGaN layer was consumed by the PEC oxidation process. Therefore, the gate-recessed regions were formed simultaneously. To obtain a stable gate oxide layer composed of mixed α-Al_2_O_3_ and β-Ga_2_O_3_, the samples were then annealed in an oxygen ambient furnace at 700 °C for 1 h to improve their condensation and quality [22]. The composition of the growth oxide layer was measured using an X-ray diffraction system. The thickness of the resulting gate oxide layer was approximately 18.0 nm, while the gate-recessed depth of the consumed AlGaN barrier layer was approximately 12.0 nm. Since the thickness of the epitaxial AlGaN barrier layer was 30.0 nm, the thickness of the remaining AlGaN barrier layer in the gate-recessed regions was 18.0 nm. To estimate the interface state density, a photoassisted capacitance–voltage (C-V) method was carried out [23]. The C-V measurement was performed at 1 MHz using an HP 4280A (Yokogawa-Hewlett-Packard, Tokyo, Japan). The estimated interface state density was approximately 5.1 × 10^11^ cm^−2^-eV^−1^ [22]. To estimate the dielectric constant of the PEC grown oxide layer, a metal-oxide-semiconductor (MOS) varactor device was designed and fabricated previously [24]. The C-V characteristics of the MOS device were measured using an HP 4280A. The estimated dielectric constant was approximately 10.6 [24]. The configuration with the PEC oxidized gate oxide layer and the source and drain electrodes is shown in Figure 1d. By using the standard photolithography method to open the gate window patterns, the Ni/Au (25/200 nm) metals were deposited on the gate oxide regions as the gate metals by an electron beam evaporator. Figure 1e and Figure 1f show the three-dimensional configuration of the GaN-based MOS-HEMTs with dual-gate and single-gate structures, respectively. Figure 2 illustrates the top view of the scanning electron microscopy image of the GaN-based DG-MOS-HEMTs. The gate length and width were 2 and 50 μm, respectively. In the DG structure, the separation between the main gate electrode and the auxiliary gate electrode was 4 μm. Additionally, the separation between the source and main electrodes, as well as between the drain and auxiliary electrodes, was 6 μm in both cases. The gate electrode of the single-gate devices was positioned in the same location as the main gate electrode of the dual-gate devices.

## 3. Experimental Results and Discussion

**Figure 3:**
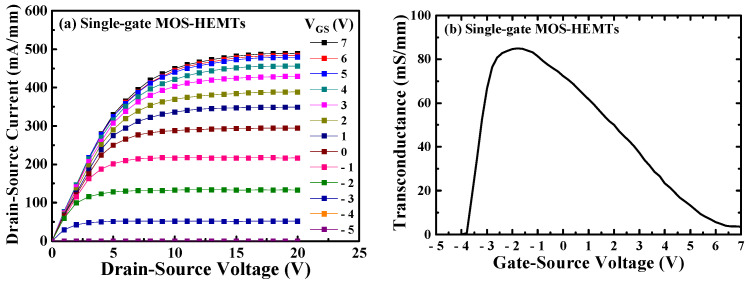
(**a**) Typical drain-source current–drain-source voltage characteristics and (**b**) typical extrinsic transconductance as a function of gate-source voltage of GaN-based single gate MOS-HEMTs.

Using the measurement of an Agilent 4156C semiconductor parameter analyzer (Keysight Technologies, Santa Rosa, CA, USA), Figure 3a shows the typical drain-source current (I_DS_)–drain-source voltage (V_DS_) characteristics of the GaN-based single-gate MOS-HEMTs. Under a V_DS_ of 20 V, by normalizing to the gate width of 50 μm, the normalized drain-source current of the single-gate devices operating at V_GS_ values of 5 V, 6 V, and 7 V was 479.2 mA/mm, 484.1 mA/mm, and 489.2 mA/mm, respectively. When the threshold voltage (V_th_) was defined as the gate voltage at which I_DS_ = 0.1 mA/mm and V_DS_ = 20 V, a V_th_ of −3.64 V was obtained. Figure 3b illustrates the extrinsic transconductance (g_m_)–V_GS_ characteristics of the GaN-based single-gate MOS-HEMTs. The maximum extrinsic transconductance (g_m,max_) was 84.9 mS/mm.

**Figure 4:**
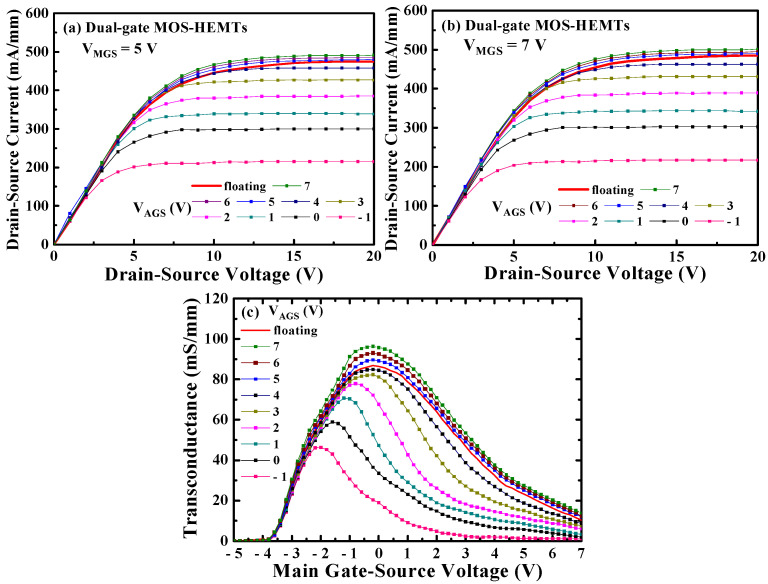
Typical drain-source current–drain-source voltage characteristics under (**a**) V_MGS_ = 5 V, (**b**) V_MGS_ = 7 V, and (**c**) typical extrinsic transconductance as a function of V_MGS_ of GaN-based dual-gate MOS-HEMTs.

To explore the advantage and influence of the auxiliary gate voltage on the characteristics of the GaN-based dual-gate MOS-HEMTs, the main gate voltage (V_MGS_) was maintained at 5 V, while the auxiliary gate voltage (V_AGS_) was kept at a floating condition and varied from −1 to 7 V in 1 V steps. To compare with the single-gate devices, Figure 4a and Figure 4b show the associated typical I_DS_-V_DS_ characteristics of the dual-gate devices operating at V_MGS_ values of 5 V and 7 V, respectively. When the V_MGS_ was 5 V and the auxiliary gate was maintained at a floating condition, the normalized drain-source current at V_DS_ = 20 V was 475.2 mA/mm, similar to the associated value of the single-gate devices. However, the associated normalized drain-source current was reduced to 215.5 mA/mm at V_AGS_ = −1 V. This phenomenon was attributed to the fact that the reverse bias voltage reduced the 2DEG density beneath the auxiliary gate region. Under the same V_MGS_ of 5 V, it was also noted that the normalized drain-source current increased with an increase in V_AGS_. Because the 2DEG density beneath the auxiliary gate region increased as the forward bias voltage increased, the drain-source current also increased. Under a V_MGS_ of 5 V, when the auxiliary gate voltage was 5 V, 6 V, and 7 V, the normalized drain-source current was 480.2 mA/mm, 485.4 mA/mm, and 491.3 mA/mm, respectively, which was larger than 479.2 mA/mm of the single-gate devices operating at a V_GS_ of 5 V. As shown in Figure 4b, it was found that the normalized drain-source current of the dual-gate devices operating at V_MGS_ = 7 V was 489.9 mA/mm, 495.1 mA/mm, and 501.4 mA/mm, which corresponded to V_AGS_ values of 5 V, 6 V, and 7 V, respectively, which was larger than 489.2 mA/mm of the single-gate device operating at V_GS_ = 7 V. It is worth noting that the dual-gate structure could modulate the drain-source current by changing the auxiliary gate voltage and increasing the drain-source current to enhance its output power. Under the operating bias voltage at various V_AGS_ voltages and V_MGS_ values of 5 V and 7 V, the threshold voltage of the dual-gate devices was maintained at −3.64 V, which was similar to that of the single-gate devices operating at V_GS_ = 5 V and 7 V. To compare with the single-gate devices, the g_m_-V_MGS_ characteristics of the dual-gate devices operating at various V_AGS_ voltages are also shown in Figure 4c. The associated g_m,max_ value was improved from 46.3 mS/mm to 89.7, 93.1, and 96.3 mS/mm when the V_AGS_ changed from −1 V to 5 V, 6 V, and 7 V, respectively. Compared with the g_m,max_ of 84.9 mS/mm of the single-gate devices, when the dual-gate devices operated at V_AGS_ = 5, 6, and 7 V, their g_m,max_ was improved. It is worth noting that the use of a dual-gate structure could not only modulate the g_m_ value by changing the V_AGS_ voltage but also improve the g_m_ value better than that of the single-gate devices.

**Figure 5:**
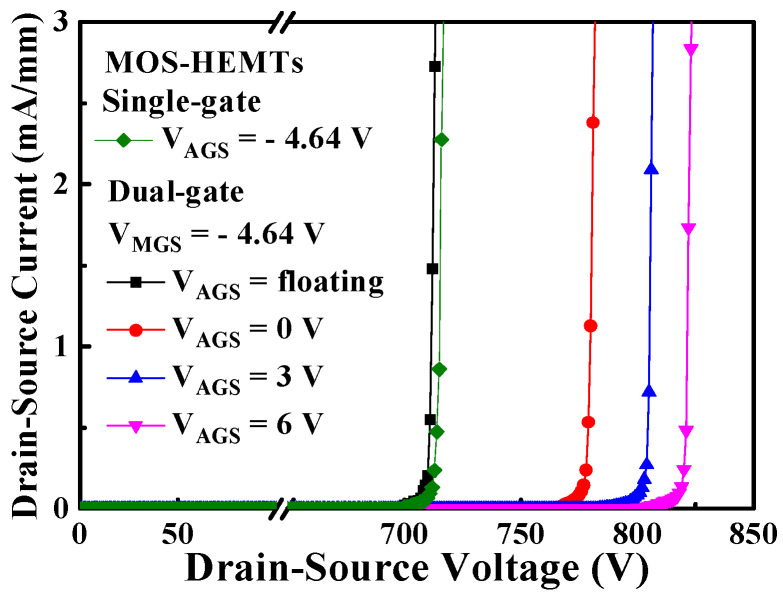
Typical drain-source current–drain-source voltage of GaN-based MOS-HEMTs with single-gate structure and dual-gate structure with various V_AGS_ voltages.

To increase the output power of GaN-based MOS-HEMTs, smoothing the electric field distribution of the 2DEG channel layer was often necessary to increase the drain-source breakdown voltage. Figure 5 shows the I_DS_-V_DS_ characteristics of the single-gate devices under the off-state operation of V_GS_ = V_th_ −1 V = −4.64 V. By defining the drain-source breakdown voltage as the voltage corresponding to I_DS_ of 1 mA/mm, it was approximately 715 V. Figure 5 shows the I_DS_-V_DS_ characteristics of the dual-gate devices under the operation of V_MGS_ = V_th_ −1 V = −4.64 V and various V_AGS_ voltages. When the V_AGS_ was at floating, 0 V, 3 V, and 6 V, the associated drain-source breakdown voltage was approximately 711 V, 780 V, 805 V, and 821 V, respectively. The drain-source breakdown voltage had a similar value for both single-gate devices and dual-gate devices operating at the floating condition of the auxiliary gate. However, the drain-source breakdown voltage was improved by incorporating an auxiliary gate and increasing the V_AGS_ voltage. When the drain-source voltage continued to rise, the attracted extra electrons in the 2DEG channel layer underneath the auxiliary gate electrode induced by the larger V_AGS_ voltage were depleted first before an electric field in the auxiliary electrode was established [25]. Consequently, the electric field distribution was smoothed while the breakdown voltage was improved accordingly.

**Figure 6:**
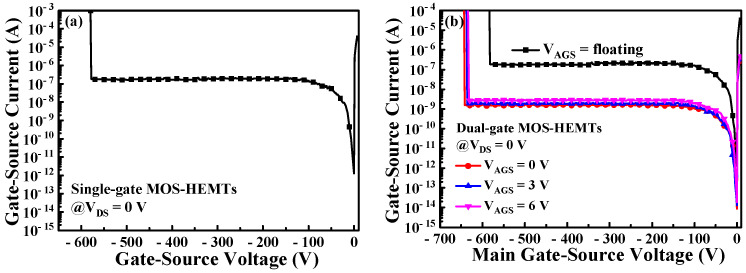
Typical gate-source current–gate-source voltage of GaN-based MOS-HEMTs with (**a**) single-gate structure and (**b**) dual-gate structure with various V_AGS_ voltages.

Under the drain-source voltage of 0 V, Figure 6a and Figure 6b show the I_GS_-V_GS_ characteristics of the single-gate devices and the I_GS_-V_MGS_ characteristics of the dual-gate devices under various V_AGS_ voltages, respectively. As shown in Figure 6a, the gate-source leakage current at V_GS_ = −100 V was 147 nA, and the maximum gate-source breakdown voltage was −580 V. The low gate-source leakage current and high gate-source breakdown voltage could verify the high quality and high insulation properties of the gate oxide layer directly grown by the PEC oxidation method. As shown in Figure 6b, for the dual-gate devices operating at V_DS_ of 0 V and various V_AGS_ voltages, the gate-source leakage current at V_MGS_ = −100 V was 153 nA, 1.13 nA, 1.42 nA, and 2.03 nA corresponding to the V_AGS_ at floating, 0 V, 3 V, and 6 V, respectively. The associated gate-source breakdown voltages were −582 V, −644 V, −638 V, and −634 V, respectively. It was found that the gate-source leakage current and gate-source breakdown voltage of the single-gate devices and the dual-gate devices operating under a floating condition of auxiliary gate exhibited similar results. However, when positive bias voltages were applied to the auxiliary gate electrode of the dual-gate devices, both gate-source leakage current and gate-source breakdown voltage were improved accordingly. In general, the auxiliary gate in the dual-gate structure capacitively couples to the 2DEG channel and modifies the lateral electric field distribution, carrier density, and current bottleneck location. This experimental result could be inferred from the fact that applying a voltage to the auxiliary gate could cause the electric field redistribution and reduce the peak electric field intensity near the main gate. However, this inference still needs to be simulated using National Taiwan University Drift Diffusion Charge Control (NTU DDCC) TCAD platform to determine its electric field distribution.

**Figure 7:**
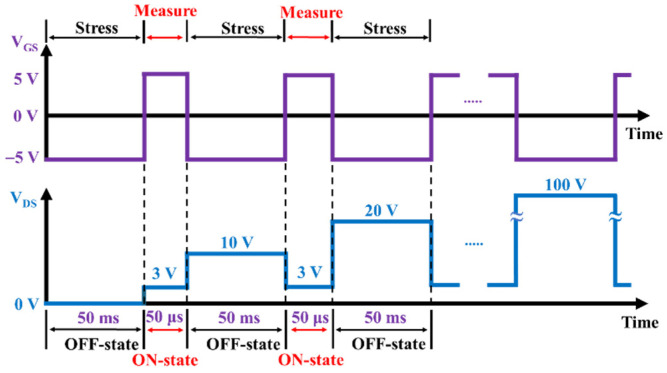
Time sequence of pulsed voltage for dynamic on-resistance measurement.

**Figure 8:**
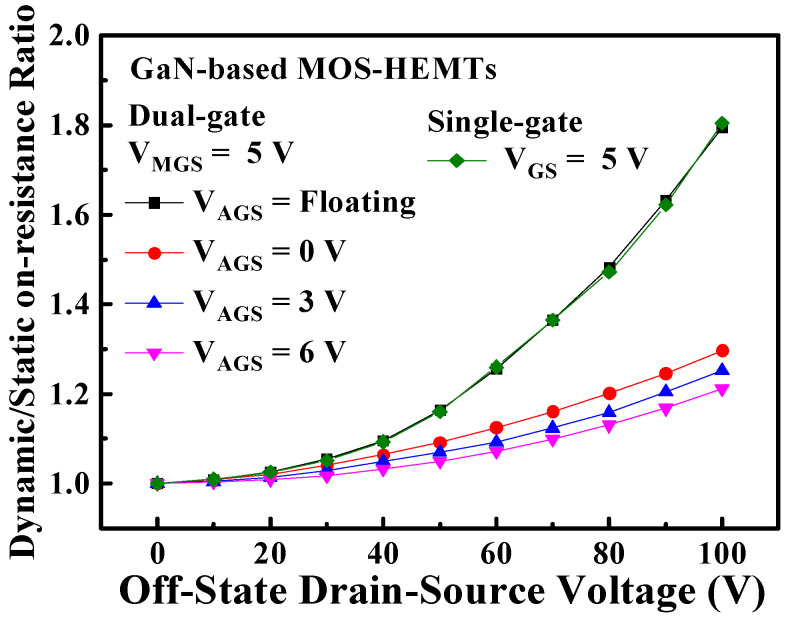
Dynamic/static on-resistance ratio of single-gate devices and dual-gate devices operating at various voltages.

To investigate the carrier trapping and detrapping behaviors of the GaN-based MOS-HEMTs under high-voltage switching operation, the dynamic on-resistance and static on-resistance were measured using an Agilent B1505A (Keysight Technologies, Santa Rosa, CA, USA). The dynamic on-resistance and the static on-resistance were defined as the resistance measured at the instant of reconduction after experiencing a voltage stress state and after the stress-free state. Figure 7 shows the voltage pulses in time series utilized in the pulsed voltage method of single-gate devices and dual-gate devices, where V_GS_ and V_MGS_ were periodically switched between −5 V (off-state, 50 ms) and +5 V (on-state, 50 μs), while V_DS_ increased in steps of 10 V from 10 V, 20 V, and finally to 100 V. The designed V_DS_ values enabled the evaluation of the different off-state voltage stress conditions. In the on state, the set of V_DS_ of 3 V ensured that the GaN-based MOS-HEMTs operated in conduction mode but did not achieve saturation mode. To verify the advantages of the auxiliary gate in dual-gate devices, the associated V_MGS_ and V_DS_ were kept the same as the V_GS_ and V_DS_ of the single-gate devices, while the V_AGS_ was set to floating, 0 V, 3 V, and 6 V, respectively. Figure 8 shows the associated dynamic/static on-resistance (D/S) ratio. Under a stress voltage of 100 V, the D/S ratio of the single-gate devices was 1.80, while the D/S ratio of the dual-gate devices operating at floating, 0 V, 3 V, and 6 V was 1.79, 1.29, 1.25, and 1.21, respectively. It was found that the D/S ratio between the single-gate devices and the dual-gate devices operating at a floating auxiliary gate was maintained at a similar value. However, by using the modulation of the auxiliary gate in the dual-gate devices, not only could the D/S ratio be reduced, but the reduction was further reduced as the V_AGS_ increased. In general, the D/S ratio revealed a degradation degree induced by carrier trapping and detrapping effects when the GaN-based MOS-HEMTs were turned back to on state after operating at a high-voltage off state. According to the experimental results of the D/S ratio, the carrier trapping and detrapping effects could be improved by the auxiliary gate modulation. The improved effects were attributed to the effective electric field redistribution and carrier accumulation in the 2DEG channel, which suppressed the probability of carrier trapping and detrapping. When a larger voltage was applied to the auxiliary gate of the dual-gate devices, a greater modulation effect occurred, and thus the carrier trapping/detrapping effect was reduced to lower the associated D/S ratio.

**Figure 9:**
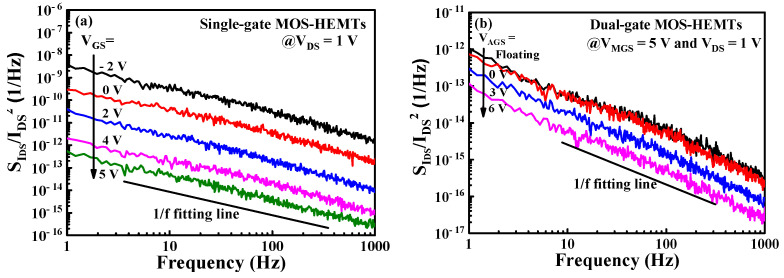
Typical frequency-dependent normalized noise power density spectra of GaN-based MOS-HEMTs with (**a**) single-gate structure and (**b**) dual-gate structure.

The Agilent 4156C semiconductor parameter analyzer (Keysight Technologies, Santa Rosa, CA, USA), HP 35670A dynamic signal analyzer (Keysight Technologies, Santa Rosa, CA, USA), and BTA 9812B (Berkeley Technology Associates, Berkeley, CA, USA) noise analyzer were used to measure the low-frequency noise power density (S_IDS_(f)) spectra. Figure 9a and Figure 9b show the typical frequency (f)-dependent normalized low-frequency noise power density spectra of the GaN-based MOS-HEMTs with single-gate and dual-gate structures operating at V_DS_ = 1 V, respectively. The normalized low-frequency power density was 3.79 × 10^−15^ Hz^−1^ for the single-gate devices operating at f = 100 Hz, V_DS_ = 1 V, and V_GS_ = 5 V, while it was 4.69 × 10^−15^ Hz^−1^, 4.12 × 10^−15^ Hz^−1^, 1.41 × 10^−15^ Hz^−1^, and 5.23 × 10^−16^ Hz^−1^ for the dual-gate devices operating at f = 100 Hz, V_DS_ = 1 V, V_MGS_ = 5 V, and V_AGS_ at floating, 0 V, 3 V, and 6 V, respectively. As shown in Figure 9a, the normalized noise power density in the single-gate devices decreased with an increase in the V_GS_ voltage. As shown in Figure 9b, under a V_MGS_ voltage of 5 V, the normalized noise power density in the dual-gate devices was decreased with increasing V_AGS_. It could also be found that the normalized noise power density had a quite well-defined tendency of 1/f. This behavior could be ascribed to the dominant noise originating from flicker noise. The experimental results indicated that the existence of trapping–detrapping centers caused by the induced defects was significantly suppressed [26,27]. In general, Hooge’s coefficient α provided a useful figure of merit for evaluating electronic devices. The α value could be calculated as follows [28]:

α = (S_IDS_(f)/I_DS_^2^)·f·(L_G_·W_G_·n_ch_·(V_GS_ − V_th_)/|V_th_|) (2)

where (S_IDS_(f)/I_DS_^2^) is the normalized noise power density, f is the frequency, L_G_ = 2 μm is the gate length, W_G_ = 50 μm is the gate width, n_ch_ = 2.0 × 10^13^ cm^−2^ is the channel sheet carrier density, V_GS_ is the gate-source voltage, and V_th_ = −3.64 V is the threshold voltage. By substituting the values of the related parameters, an α value of 1.79 × 10^−5^ was obtained for the single-gate devices operating at f = 100 Hz, V_DS_ = 1 V, and V_GS_ = 5 V. Under the operating conditions of dual-gate devices at f = 100 Hz, V_DS_ = 1 V, and V_MGS_ = 5 V, the α values were 2.23 × 10^−5^, 1.96 × 10^−5^, 6.67 × 10^−6^, and 2.47 × 10^−6^ corresponding to V_AGS_ at floating, 0 V, 3 V, and 6 V, respectively. From the experimental results of the normalized noise power density and the corresponding α value, the noise performance was improved by adding an auxiliary gate in the dual-gate devices. The noise improvement was attributed to the fact that applying positive bias voltages to the auxiliary gate electrode could concentrate the added electrons into the 2DEG channel to further prevent the electrons from being trapped and detrapped by the traps and defects of the GaN-based epitaxial layers, thereby suppressing the noise interference caused by traps and defects [29].

## 4. Conclusions

To emphasize the influence of the dual-gate structure in GaN-based depletion-mode MOS-HEMTs, interface states and surface damage were minimized using the PEC oxidation method to directly grow a gate oxide layer and simultaneously create gate-recessed regions. Compared with the single-gate structure, in addition to being modulated by the main gate, the dual-gate devices’ electrons in the 2DEG channel layer were also modulated by the auxiliary gate. By modulating the electron density in the 2DEG channel layer with the help of the auxiliary electrode, the main gate could modulate a broader range of drain-source current. Therefore, a larger transconductance could also be obtained. Under a drain-source voltage of 20 V, a drain-source current of 479.2 mA/mm and 489.2 mA/mm was obtained for the single-gate devices operating at a gate-source voltage of 5 V and 7 V, respectively. Under a V_AGS_ of 7 V, when the dual-gate devices operated at V_MGS_ = 5 V and 7 V, their drain-source current was 491.3 mA/mm and 501.4 mA/mm, which was larger than the I_DS_ value of the single-gate devices operating at V_GS_ values 5 V and 7 V, respectively. Furthermore, compared with the g_m,max_ of 84.9 mS/mm of the single-gate devices, the g_m,max_ was improved to 96.3 mS/mm when the dual-gate devices operated at V_AGS_ = 7 V. Additionally, since applying a larger V_AGS_ voltage would deplete the attracted extra electrons underneath the 2DEG channel layer of the auxiliary gate electrode before establishing an electric field in the auxiliary electrode, smoothing the electric field distribution helps to improve the source-drain breakdown voltage. Although field plate engineering could be utilized to enhance breakdown voltage [30,31], the dual-gate structure would be a promising alternative configuration in GaN-based MOS-HEMTs. Similarly, the gate leakage current and gate breakdown voltage were also significantly improved with the addition of an auxiliary gate in the dual-gate devices. Furthermore, by applying a positive bias voltage to the auxiliary gate electrode, the attracted extra electrons in the 2DEG channel could prevent the electrons from being trapped and detrapped by the traps and defects residing in the epitaxial layers. Consequently, the noise characteristics of the dual-gate devices could be improved due to the suppression of noise interference induced by traps and defects. The high-frequency characteristics are very important for estimating the performance of devices. However, the high-frequency measurement was not carried out in the study because the dimension of the fabricated devices did not meet the probe dimension used for measuring high-frequency behaviors. Table 1 presents a quantitative comparison of the GaN-based MOS-HEMTs (HEMTs) with single-gate and dual-gate structures [15,18,32,33,34,35]. Using the PEC oxidation method to directly grow a gate oxide layer and simultaneously create gate-recessed regions, it is expected that the performance can be further improved by accurately simulating and designing to optimize the dimensions and structure of GaN-based dual-gate MOS-HEMTs.

## Figures and Tables

**Table 1:** Quantitative comparison of the GaN-based MOS-HEMTs (HEMTs) with single-gate and dual-gate structures.

Type	V_th_ (V)	I_DSS_ (mA/mm)	g_m,max_ (mS/mm)	Breakdown Voltage (V)	S_IDS_/I_DS_^2^ (Hz^−1^)	Ref.
Dual gate	−4.4	615 @V_GCS_ = 2 V, V_GCG_ = 0 V, V_DS_ = ~10 V	183 @V_GCG_ = 0 V, V_DS_ = 10 V	116 @V_GCS_ = −6 V, V_GCG_ = 0 V I_DS_ = 1 mA/mm	-	[15]
Dual gate	−3.76	368 @V_MGS_ = V_AGS_ = 2 V, V_DS_ = ~6 V	-	1129 @V_MGS_ = −10 V V_AGS_ = 0 V, I_DS_ = 1 mA/mm	-	[18]
Dual gate	−4.8	500 @V_GS-DC_ = 3 V, V_GS-RF_ = 0 V, V_DS_ = ~5 V	125 @V_GS-DC_ = 3 V, V_DS_ = ~5 V	-	-	[32]
Dual gate	−2.8	429 @V_GS1_ = V_GS2_ = 4 V, V_DS_ = ~10 V	79 @V_GS1_ = −1 V, V_DS_ = 15 V	416 @off-state condition	-	[33]
Dual gate	−6	800 @V_G1S_ = 0 V, V_G2S_ = 2 V, V_DS_ = ~6 V	220 @V_G2S_ = 2 V, V_DS_ = ~6 V	-	-	[34]
Dual gate	−3.5	1179 @V_GS1_ = 2 V, V_GS2_ = 0 V, V_DS_ = 10 V	-	-	-	[35]
Single gate	−3.64	489.2 @V_GS_ = 7 V, V_DS_ = 20 V	84.9 @V_DS_ = 20 V	715 @ V_GS_ = −4.64 V, I_DS_ = 1 mA/mm	3.79 × 10^−15^ @V_DS_ = 1 V, V_GS_ = 5 V	This work
Dual gate	−3.64	501.4 @V_MGS_ = 7 V, V_AGS_ = 7 V, V_DS_ = 20 V	96.3 @V_AGS_ = 7 V, V_DS_ = 20 V	821 @V_MGS_ = −4.64 V, V_AGS_ = 6 V, I_DS_ = 1 mA/mm	5.23 × 10^−16^ @V_MGS_ = 5 V, V_AGS_ = 6 V, V_DS_ = 1 V	This work

## Data Availability

The data presented in this study are available upon request from the corresponding author.
